# Histopathology of Post-Transplant Liver Biopsies, the First Report From Iran

**DOI:** 10.5812/hepatmon.9389

**Published:** 2013-06-18

**Authors:** Bita Geramizadeh, Dorna Motevalli, Saman Nikeghbalian, Seyed Ali Malek Hosseini

**Affiliations:** 1 Transplant Research Center, Shiraz University of Medical Sciences, Shiraz, IR Iran; 2 Department of Pathology, Shiraz University of Medical Sciences, Shiraz, IR Iran; 3 Department of Surgery, Transplant Center, Shiraz University of Medical Sciences, Shiraz, IR Iran

**Keywords:** Liver Transplantation, Biopsy, Iran

## Abstract

**Background:**

Evaluation of a transplanted liver by Imaging techniques and enzyme changes is sensitive to hepatocellular or biliary problems, but in most instances liver allograft biopsies are performed in order to find out the final reason for these changes.

**Objectives:**

It’s been about 17 years (with more than 1326 cases) since the first liver transplantation in the Namazi Hospital of Shiraz University of Medical Sciences while during the last five years the number of post liver transplant biopsies have increased. Until now there has been no report of the pathological results of post liver transplant needle biopsies from Iran.

**Materials and Methods:**

During the last 5 years, there have been 382 post liver transplant biopsies. We studied the clinical charts and pathological results of all needle biopsies.

**Results:**

A total of 382 needle biopsies were performed on 287 patients aged between 1 and 64 years old. The earliest specimen was obtained within the first few hours following transplantation, and the last was gathered 3209 days (261 ± 523) post-transplantation. Acute rejection was the most common diagnosis, which occurred in 180 (47%) of specimens. Among other complications were vascular problems (8.6%), preservation/reperfusion (I/R) injury (7%), chronic rejection (5.2%), biliary injury/obstruction (3.4%), recurrence of primary disease (2.6%), drug-induced hepatic injury (1.8%), cirrhosis (1.6%), sepsis (1.4%), cytomegalovirus hepatitis (1.4%), post-transplantation lymphoproliferative disease (1%) and Venous outflow obstruction (0.5%).

**Conclusions:**

The most common pathological diagnosis of post-transplant liver needle biopsies has been acute rejection, followed by ischemia due to hepatic artery thrombosis, preservation/reperfusion injury, and chronic rejection.

## 1. Background

Liver transplantation has been accepted as the main therapeutic option for patients with end-stage liver diseases (1). Monitoring of the transplanted liver by Imaging techniques and enzyme changes are sensitive to hepatocellular or biliary injuries and problems, but in most cases, liver allograft biopsies are performed in order to find out the reason for these changes (2, 3). Therefore, in many instances liver histology remains the "gold standard" for the diagnosis of allograft dysfunction (4). The following is a review of pathology in 382 needle biopsies of 287 liver allograft recipients. This is the first report of post liver transplant biopsies from Iran.

## 2. Objectives

It’s been about 17 years (with more than 1326 cases) since the first liver transplantation in the Namazi Hospital of Shiraz University of Medical Sciences while during the last five years the number of post liver transplant biopsies have increased. Until now there has been no report of the pathological results of post liver transplant needle biopsies from Iran. Therefore in this study we are trying to present our experience regarding the results of post liver transplantation biopsies in our center as the largest center in Iran.

## 3. Materials and Methods

This study was conducted from December 2005 to January 2011 in the Department of Pathology, Namazi Hospital, Shiraz, Iran. Information including age, reason for transplantation, date of biopsies and the pathological report including the main cause of graft injury were collected, using pathology files and patients’ clinical charts. All patients undergoing needle biopsies post-transplantation in our center were considered for the study. A total of 382 needle Biopsies were obtained from 287 patients, few hours to 3209 days after transplantation. Blind or Ultra-sound guided liver needle biopsy was obtained. Specimens were fixed in 10 % buffered formaldehyde and embedded in paraffin. Seven consecutive sections were cut at a thickness of 3 micrometers from paraffin-embedded biopsies. Biopsy sections were stained by hematoxylin–eosin (H&E) methods, Masson’s trichrom, reticulin, periodic acid–Schiff with diastase, prussian blue, orcein and again with H&E. Immunohistochemical studies were done if needed such as CK19 (Cytokeratin 19), CMV (Cytomegalovirus), HBV (Hepatitis B virus), and EBV (Epstein Barr virus). The following features were evaluated in liver biopsies:

1) Number of portal tracts and adequacy of the biopsy (Biopsies with at least 5 portal tracts were considered adequate).

2) Architecture of the liver (both lobular and vascular)

3) Presence of any pathology including acute and chronic rejections, infection, malignancies, etc.

Grading of the disease accordingly, for example for acute rejection grading was made based on the BANFF 1997 (5) criteria. Criteria for evaluating chronic rejection was based on Banff Schema published in 2000 (6). Statistical Analysis was performed by using Microsoft Excel software for Windows.

## 4. Results

**Figure 1.**
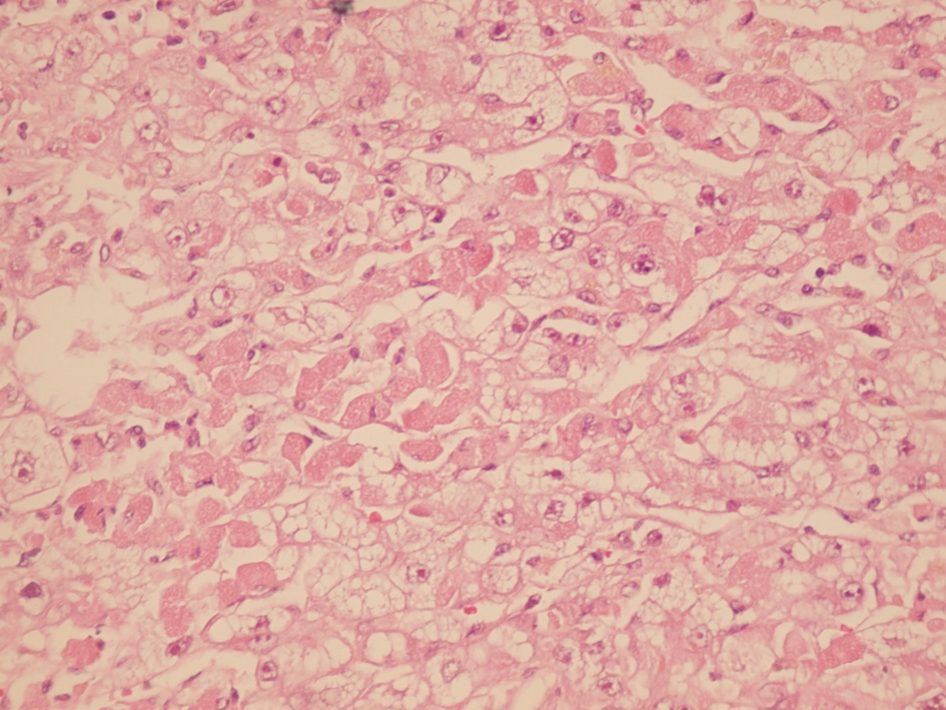
Sections From Liver Needle Biopsy Show Pericentral Ballooning Degeneration and Single Cell Apoptosis, 7 Days Post Transplantation (H&E X250)

**Figure 2.**
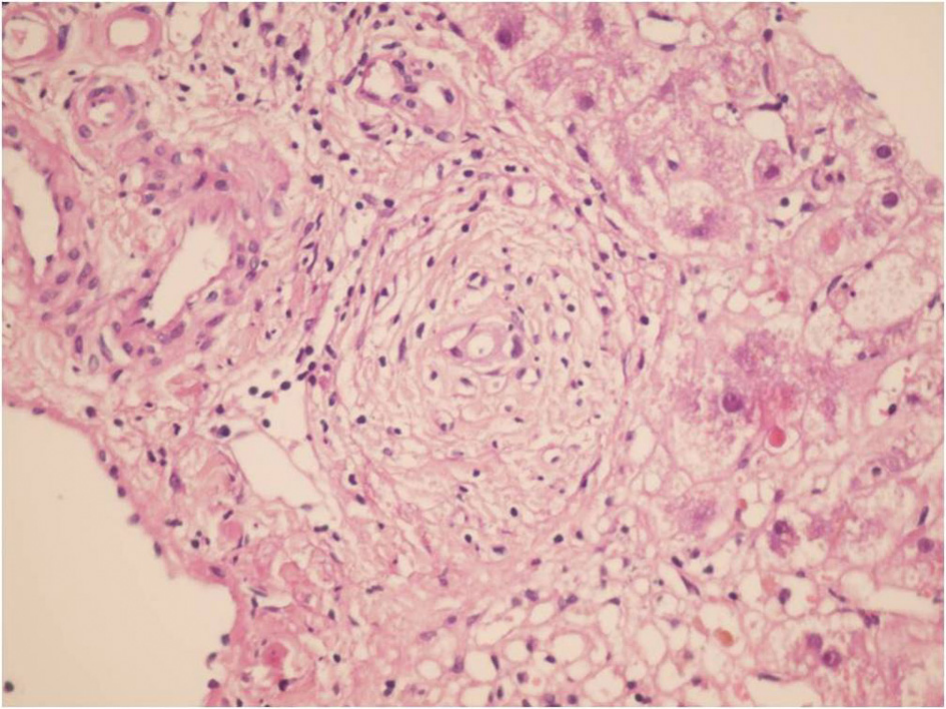
Sections From Liver Needle Biopsy Show Marked Bile Duct Epithelial Damage, 3 Months Post Transplantation, Which is considered as Early Chronic Rejection (H&EX250)

**Figure 3.**
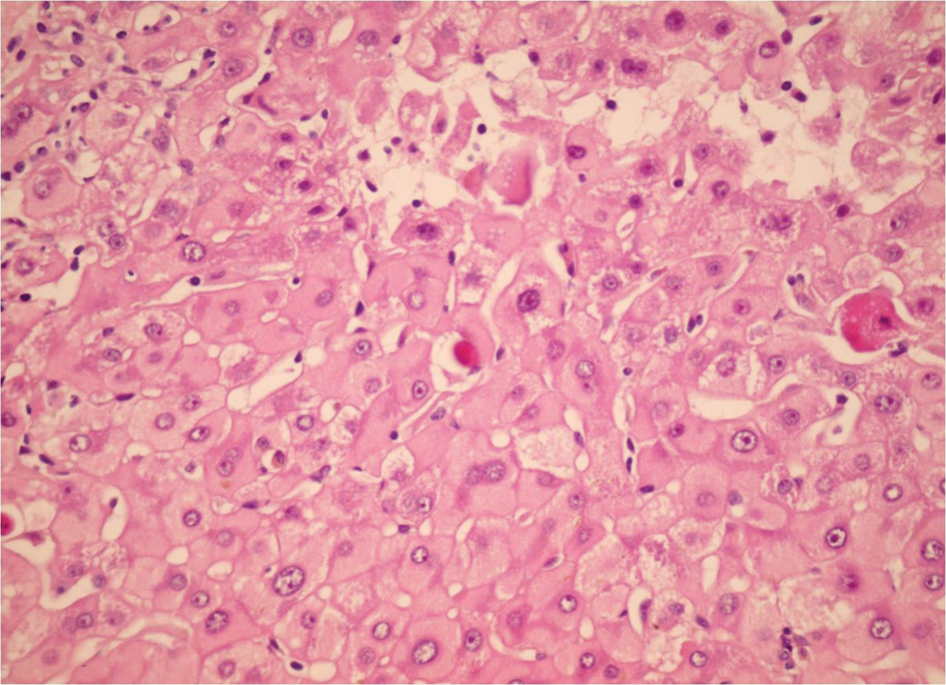
Sections From Liver Needle Biopsy in a Recurrent Chronic Hepatitis B Shows Ground Glass Hepatocytes (H&E X250)

**Figure 4.**
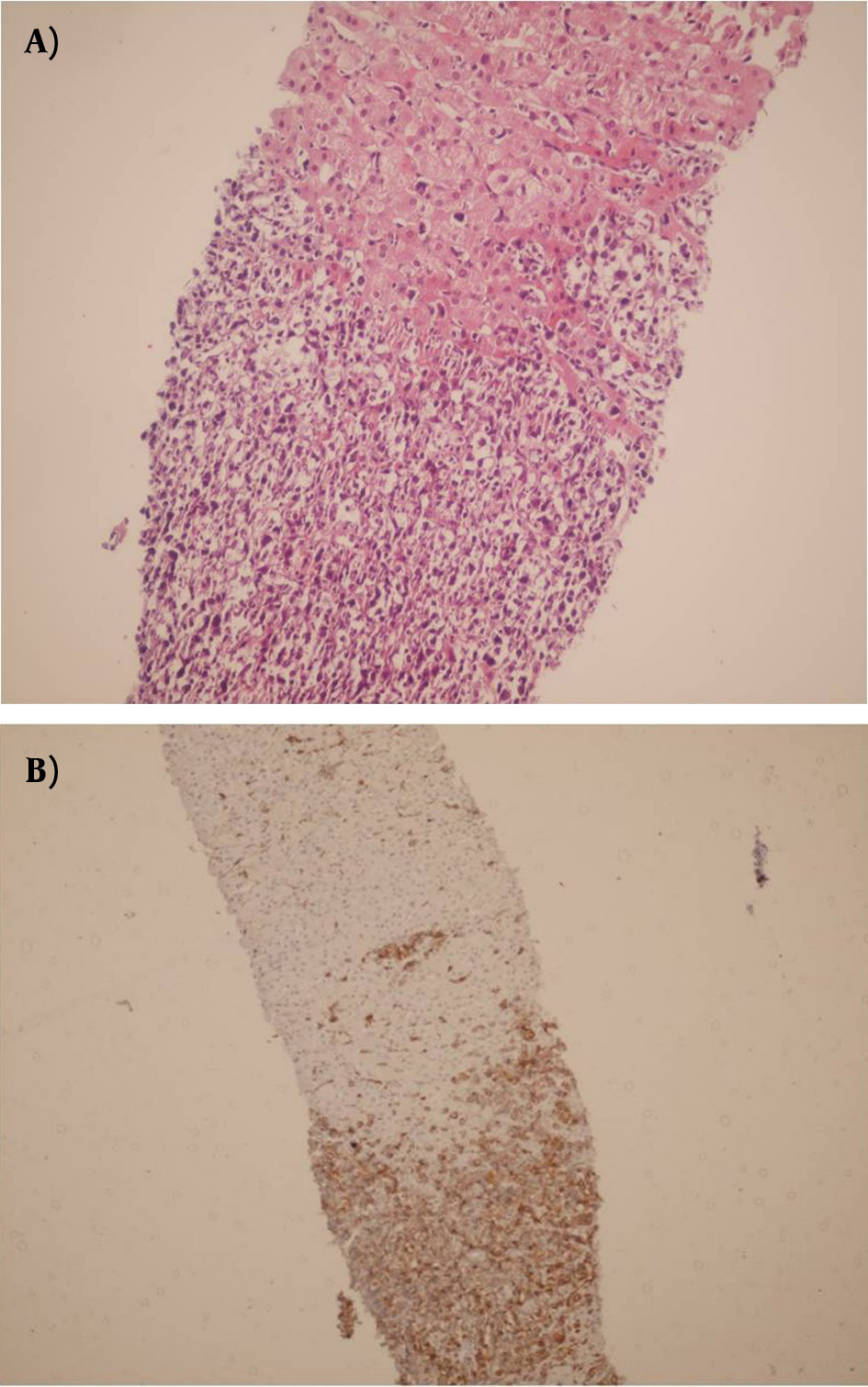
Sections From Allografted Biopsy Shows Dense Infiltration of Lymphoma Cells, Diagnosed as PTLD (A and B) (a: H&EX250, b:IHC for CD20 )

During the study period (2005-2011) a total of 382 indicated needle biopsies were obtained from 287 patients. The age of the patients was 1-64 years (28.6 ± 16.36). The most common indication of transplantation in our center was Hepatitis B (20.2%), however less common causes were autoimmune hepatitis, primary sclerosing cholangitis etc. In this study, the earliest specimen was obtained within a few hours following transplantation, and the latest was gathered 3209 days (261 ± 523) days post-transplantation (Table 1). These cases were among the 1326 liver transplants performed during the past 17 years (Table 2). Acute rejection was the most common diagnosis; 180 (47%) of 382 liver biopsies were in favor of acute rejection. Ischemic changes, including mild ischemia to severe zone 3 necrosis and complete coagulative necrosis occurred in 33 liver biopsies among this group, these changes were detected from 0 to 2676 days post- transplantation (163 ± 498.9). The cause of these ischemic changes was hepatic artery thrombosis. Preservation/reperfusion injury were mostly detected soon after transplantation, which ranged from 0 to 17 days (6.1 ± 5.5) following transplantation in 8.6% of biopsies (Figure 1). Chronic Rejection (CR) mainly occurred late in the post-transplantation period. In Our study CR was detected in 20 (5.2%) of the liver biopsies and time of occurrence was from 90 to 3128 days after transplantation (88.7 ± 865) (Figure 2). Large bile duct obstruction was diagnosed in 13 (3.4%) specimens from 0 to 17 days (45.2 ± 58.6%). Recurrence of hepatitis happened in 10 cases (2.6%) during 233 to 1257 days following transplantation. Of these cases, 3 patients were diagnosed with hepatitis B and in the remaining 7 patients, recurrent hepatitis C was diagnosed. All of the hepatitis B recurrences were among the patients who had not received immunoglobulin and antiviral agents properly. All of the 3 cases of recurrent hepatitis B were confirmed by immunohistochemistry with surface antigen (HBS Antigen) and viral load (Figure 3). Cases suspicious to recurrent hepatitis C were also confirmed by viral load. The incidence of cirrhosis was 1.6% in allograft biopsies, which occurred in 6 patients, from 390 to 3126 days post-transplantation (1200 ± 929.8). These six cases were patients with previous diagnosis of autoimmune hepatitis (3 patients) and hepatitis C related cirrhosis (3 patients). Post-transplant lymphoproliferative disorder (PTLD) had also been diagnosed in allografted liver of 4 patients as part of a systemic disease or as a primary site of involvement (Figure 4). Drug-induced cholestasis due to antibiotics such as sulfamethoxazole-trometoprime and amoxicillin were also detected in 7 patients after liver transplantation. Other rare diagnoses were infections due different viral agents such as CMV and tuberculosis.

**Table 1.** Frequency and Timing of Different Histopathologic Findings in 382 Patients

Diagnosis	Biopsies, No. (%)	Mean Days	SD	Minimum days post-Transplant	Maximum days post-Transplant
**Acute Rejection**					
Borderline (RAI ^a^ = 1)	10 (2.6)	346.4	418.0	12	1351
Mild (RAI = 2, 3)	93(24.3)	148.3	360.2	0	2210
Moderate (RAI = 4, 5, 6)	33(8.6)	326.1	650.4	0	3209
Severe (RAI = 7, 8, 9)	44(11.5)	310.5	524.3	0	2616
**Ischemic changes (Hepatic artery thrombosis)**	33(8.6)	163.0	498.9	0	2676
**Preservation/reperfusion injury**	27 (7)	6.1	5.5	0	17
**Chronic rejection**	20 (5.2)	887.7	865.0	90	3128
**biliary obstruction**	13(3.4)	45.2	58.6	0	176
**Recurrence** **of primary disease**	10 (2.6)	672.8	349.6	233	1257
**Drug induced cholestasis**	7 (1.8)	229.4	237.6	14	690
**Post liver ** **Tx** **^a^** ** cirrhosis**	6 (1.6)	1200	929.8	390	3126
**Sepsis **	5 (1.4)	111.8	136.3	3	364
**CMV ** **^a^** ** Hepatitis**	5 (1.4)	23.4	12.5	7	43
**PTLD ** **^a^** ** in allograft liver**	4 (1)	715	594.7	171	1700
**Venous Outflow obstruction**	2 (0.5)	1.5		1	2
**TB ** **^a^** ** in ** **allografted** ** liver**	1 (0.26)	1818			
**Suboptimal **	2 (0.5)				
**Normal pathology**	67 (17.74)				

^a^ Abbreviations: RAI, Rejection activity index; PTLD, Post-transplant lymphoproliferaive Disorder; TX, transplant; TB, Tuberculosis

**Table 2.** The Causes of Liver Transplantations, Performed Before January 2012 in Shiraz Transplant Center Among 1326 Patients

Cause of Transplant	Number	Percentage, %
**Hepatitis B**	290	21.8
**Autoimmune hepatitis**	235	17.7
**Primary ** **Sclerosing** ** cholangitis**	150	11.5
**Wilson**	115	6.7
**Biliary atresia**	60	5.6
**Tyrosinemia**	40	3
**Hepatitis C**	40	3
**Progressive familial intrahepatic cholestasis**	40	3
**Primary biliary cirrhosis**	18	1.5
**Budd-** **Chiari**	30	2.4
**Crigler-Najjar**	30	2.4
**Hypercholestrolemia**	25	2
**Alcoholic**	15	1.5
**Neonatal hepatitis**	9	0.6
**Acute liver failure**	6	0.45
**Caroli**	5	0.4
**Hyperoxaluria**	3	0.3
**Congenital hepatic fibrosis**	4	0.3
**Neoplastic**	11	0.8
**Cryptogenic (NASH)**	200	15

## 5. Discussion

Liver allograft pathology continues to play an important role in the diagnosis and management of complications in the course of liver transplantation. Liver needle biopsy is a convenient method for monitoring allograft complications (7). Histopathological assessments continue to play an important role in the diagnosis and management of liver allograft rejection. Changes that occur in acute and chronic rejection are well recognized and liver biopsy remains the “gold Standard” for diagnosing these two conditions (8). In our study the most common histopathological finding in post liver transplant biopsies was acute rejection occurring in 46% of the cases, most of which were mild acute rejection i.e. RAI (rejection activity index) was below 3. This finding was similar to that reported by previous studies (9, 10). Acute rejection occurring after 6 months from transplantation has been termed, late acute rejection by some authors (8). We had the same experience with acute rejections, 6 months post transplantation where the histological features of rejection had changed with time. The gold standard for the diagnosis of chronic rejection is also based on liver biopsy findings, and the incidence of this condition has been reported to be between 2 and 20% (2, 8).

According to our experience, the incidence of chronic rejection in our center was about 5.2%, and we had chronic rejections as early as 3 months post liver transplantation. More time is required to decide the exact rate of chronic rejection in our center. The other major concern in 7% of our cases was preservation/reperfusion injury in the first 2 weeks post liver transplantation using deceased donors’ livers. In other studies this condition has been reported in about 13.4% of transplanted livers (9). Our results showed better preservation and less hepatic damage during cold and warm ischemic times. Post liver transplantation fibrosis, and cirrhosis have been reported in previous studies in 10-30% of liver transplant patients, mostly in the patients with underlying chronic hepatitis such as hepatitis C and autoimmune hepatitis related cirrhosis as early as 18 months (11, 12). This was much less in our experience i.e. we had post liver transplant cirrhosis in only 6 patients (1.6%) and as early as 15 months in a patient who had been transplanted due to hepatitis C related cirrhosis. The lower incidence of post liver transplant cirrhosis might be because of shorter duration of follow up in our patients (it is only 17 years after the first liver transplant in our center) and also less hepatitis C related cirrhosis in our transplanted patients compared with western countries. Hepatic artery thrombosis and different degrees of ischemic hepatocyte necrosis in our cases have been present in 8.6% of post liver transplant biopsies. This incidence in previous reports has been 1.6 to 10.5%, as early as less than one hour post transplantation (13, 14). Long-term survival after liver transplantation due to improvements in surgical techniques, and immunosuppressive regimens, has increased the recurrence rate of the primary diseases. The frequency of recurrence depends on the etiology and the primary liver disease (2). Regarding the recurrence of hepatitis B, currently combination therapy of oral antiviral agents and hepatitis B immunoglobulin in the pre and post-transplant period has achieved complete protection (15). However, there were 3 patients with recurrent hepatitis B in our study, because the patients hadn’t received their regimen properly due to incompliance. The remaining recurrent diseases were HCV chronic hepatitis. According to infectious diseases, we’ve had rare CMV hepatitis involving allografted livers. CMV hepatitis is a significant complication after liver transplantation and has a reported incidence of 2-10% (16). In the period of this study we experienced 5 cases of CMV hepatitis documented by liver biopsy, which were confirmed by immunohistochemistry. We had an unusual case of post liver transplant tuberculosis with the isolated involvement of the allografted liver which was treated successfully and has been described in details in another paper in press (17). Post-transplant lymphoproliferative disorder (PTLD) has been diagnosed in 4 cases of allografted livers. Diagnosis of PTLD in the allografted liver is not common and can be part of a pre-existing PTLD, but rarely can be the primary manifestation of the disease (18). In this series of transplanted patients, two PTLD of the allografted liver were primary and in another two liver involvement was part of the systemic disease (19). We diagnosed 7 cases with drug-induced cholestasis. In the majority, antibiotics such as amoxicillin and sulfamethoxazole were considered as the probable cause and discontinuation resulted in recovery and this has been reported by previous studies (20, 21). We had a case of cholestasis and severe pruritis about 2 years post transplantation after use of anabolic steroid for body-building in a patient with previous diagnosis of primary sclerosing cholangitis; the patient’s condition improved with discontinuation of the drug. Technical complications such as biliary stricture of the anastomosis site and vascular complications have also been rarely diagnosed in our patients during the last 5 years. In conclusion, our hospital is the main liver transplant center in Iran and also the majority of post-transplant liver biopsies are performing here (It is worthy to note that nearly all liver biopsies taken outside of our center are reviewed as a consult case and has been included in this study), thus we have experienced all the reported complications resulting from liver biopsies.
